# rCUR: an R package for CUR matrix decomposition

**DOI:** 10.1186/1471-2105-13-103

**Published:** 2012-05-17

**Authors:** András Bodor, István Csabai, Michael W Mahoney, Norbert Solymosi

**Affiliations:** 1 Department of the Physics of Complex Systems, Eötvös Loránd University, Pázmány Péter sétány 1/A, 1117, Budapest, Hungary; 2 Department of Mathematics, Stanford University, Stanford, CA 94305, USA; 3 Department of Animal Hygiene, Herd Health and Veterinary Ethology, Szent István University, István utca, 1078, Budapest, Hungary

## Abstract

**Background:**

Many methods for dimensionality reduction of large data sets such as those generated in microarray studies boil down to the Singular Value Decomposition (SVD). Although singular vectors associated with the largest singular values have strong optimality properties and can often be quite useful as a tool to summarize the data, they are linear combinations of up to all of the data points, and thus it is typically quite hard to interpret those vectors in terms of the application domain from which the data are drawn. Recently, an alternative dimensionality reduction paradigm, *CUR matrix decompositions*, has been proposed to address this problem and has been applied to genetic and internet data. CUR decompositions are low-rank matrix decompositions that are explicitly expressed in terms of a small number of actual columns and/or actual rows of the data matrix. Since they are constructed from actual data elements, CUR decompositions are interpretable by practitioners of the field from which the data are drawn.

**Results:**

We present an implementation to perform CUR matrix decompositions, in the form of a freely available, open source R-package called rCUR. This package will help users to perform CUR-based analysis on large-scale data, such as those obtained from different high-throughput technologies, in an interactive and exploratory manner. We show two examples that illustrate how CUR-based techniques make it possible to reduce significantly the number of probes, while at the same time maintaining major trends in data and keeping the same classification accuracy.

**Conclusions:**

The package rCUR provides functions for the users to perform CUR-based matrix decompositions in the R environment. In gene expression studies, it gives an additional way of analysis of differential expression and discriminant gene selection based on the use of statistical leverage scores. These scores, which have been used historically in diagnostic regression analysis to identify outliers, can be used by rCUR to identify the most informative data points with respect to which to express the remaining data points.

## Background

In many modern data analysis applications, the user is faced with data matrices with a huge number of columns and/or rows. Such matrices arise in disciplines ranging from astronomy through genomics and social sciences to zoology. As a specific example, let us consider gene expression microarray data. In a typical study, hundreds of thousands of probe expressions are measured for a large number of samples. This methodology has had a significant impact on gene expression research, but the publication of studies with dissimilar or contradictory results has raised concerns about the reliability of this technology, especially when all the individual values of gene expressions are requested. On the other hand, when the goal is more modest, *e.g.*, just classifying the samples into few categories, there is typically ample information available in the data, and one can hope that the huge redundancy in the data compensates for the possible errors of the technology.

In such cases, it is common to employ one of several dimensionality reduction methods in order to identify low-dimensional features for use by a downstream analyst. Many popular methods, *e.g.*, Principal Component Analysis (PCA), multidimensional scaling, recently-popular nonlinear-dimensionality reduction methods, etc., boil down to the Singular Value Decomposition (SVD). The singular vectors, or principal components, associated with the largest singular values have strong optimality properties, and they can often be quite useful as a tool to summarize and identify major patterns of the data. (See, e.g., [1], as a nice example in the field of genomics and [2] for a fast matrix factorization algorithm.) Nevertheless, it is typically quite hard for a geneticist or downstream data analyst to interpret those vectors in terms of the application domain from which the data are drawn. The reason for this is that the singular vectors are mathematical abstractions defined for any matrix, and they are typically linear combinations of all of the input data. This has been noted most explicitly by Kuruvilla *et al.*[3]. After describing the many uses of the vectors provided by the SVD and PCA in DNA microarray analysis, they bluntly conclude that "While very efficient basis vectors, the (singular) vectors themselves are completely artificial and do not correspond to actual (DNA expression) profiles. .. Thus, it would be interesting to try to find basis vectors for all experiment vectors, using actual experiment vectors and not artificial bases that offer little insight."

To address these and other issues, Mahoney and Drineas [4] proposed the *CUR matrix decomposition* method. CUR decompositions are low-rank matrix decompositions that are explicitly expressed in terms of a small number of actual columns and/or actual rows of the data matrix:

(1) A≈CUR

where *A* is the original data matrix, *C* consists of a small number of actual columns of *A*, *R* consists of a small number of actual rows of *A*, and *U* is a small carefully constructed matrix that guarantees that the product *CUR* is close to *A*. Since they are constructed from actual data elements, CUR decompositions are interpretable by practitioners of the field from which the data are drawn (to the extent that the original data are). For example, CUR decompositions have been used for interpretable data analysis of DNA single-nucleotide polymorphism data [5-7]. The theory of CUR matrix decompositions works as follows [4,8]. To determine which columns to include in *C* (and similarly for *R*), one computes an "importance score" for each column of *A* and then randomly samples a small number of columns from *A* using that score as an "importance sampling" probability distribution. This importance score depends on the matrix *A* and an input rank parameter *k*. If $v_{j}^{\xi }$ is the *j*-th element of the *ξ*-th right singular vector of *A*, then the *normalized statistical leverage scores* equal

(2) $$\;\Pi_{j}=\frac{1}{k}\overset{k}{\underset{\xi =1}{\sum }}{\left(v_{j}^{\xi }\right)}^{2},$$

for all *j *= 1,…,*n*. These quantities, up to scaling, equal to the diagonal elements of the so-called "hat matrix," *i.e.*, the projection matrix onto the span of the top *k* right singular vectors of *A*[9]. As such, they have a natural statistical interpretation as a "leverage score" or "influence score" associated with each of the data points; and they have been widely-used for outlier identification in diagnostic regression analysis.

The basic algorithm for choosing columns from a matrix—call it ColumnSelect—takes as input *any m *×* n *matrix *A*, a rank parameter *k*, and an error parameter *ε*, and then performs the following. 

1. First, compute *v*^1^,…,*v*^*k*^(the top *k* right singular vectors of *A*) and the normalized statistical leverage scores of Equation (2).

2. Second, keep the *j*-th column of *A* with probability $p_{j}=\text{min}\{1,c\Pi_{j}\}$, for all *j *∈ {1,…,*n*}, where $c=O(k\text{log}k/\varepsilon^{2})$.

3. Third, return the matrix *C* consisting of the selected columns of *A*.

In some applications, this restricted CUR decomposition, *A *≈* P*_*C*_*A *=* CX*, where *X *=* C*^ + ^*A*, is of interest and where *C*^ + ^denotes a Moore-Penrose generalized inverse of the matrix *C*.^a^

In other applications, one wants a CUR matrix decomposition in terms of columns and rows simultaneously. The basic algorithm for this performs the following. 

1. Run ColumnSelect on *A* with $c=O(k\text{log}k/\varepsilon^{2})$ to choose columns of *A* and construct the matrix *C*.

2. Run ColumnSelect on *A*^*T *^with $r=O(k\text{log}k/\varepsilon^{2})$ to choose rows of *A* (columns of *A*^*T*^) and construct the matrix *R*.

3. Define the matrix *U* as *U *=* C*^ + ^*AR*^ + ^.

Thus, in contrast to PCA and the SVD, where the low-dimensional basis consists of singular vectors that are linear combinations of all the data vectors, here the matrices *C* and *R* consists a small number of actual columns and rows of *A*, respectively. The details of this procedure, including the use of randomness, are important for the strong underlying theory [4,8,10]; but in practice several variations that exploit the structure identified by the statistical leverage scores perform very well. These practical design decisions we made for our implementation will be described in the next section.

In this paper, we describe the rCUR package, which is a freely available, open source R implementation of the CUR matrix decomposition method. We will summarize functionality and features of the package that allow the user to obtain the statistical leverage scores and the matrices *C*, *U*, and *R* by simple S4 classes and methods. In certain cases, we have found that the statistical leverage scores themselves are useful directly, and thus we also describe variations to select the columns or rows that deviate from the theory described above. We will then demonstrate the strength of the technique on a microarray study. In particular, we will show that even for a very large set of heterogeneous samples with various experiments, rCUR is able to select a few percent of the probes that have the same classification capacity as the original full set.

Finally, it should be emphasized that this CUR approach is very different the classical statistical perspective, where statistical leverage scores have been used in diagnostic regression analysis to identify outliers and errors [9]; and that Bien *et al.*[11] have described the connections between CUR matrix decompositions and sparse optimization methods. See also [12] as a previous example in the genomics literature for gene selection via outliers.

## Implementation

The rCUR package was developed to allow users to easily perform CUR matrix decompositions. For this purpose, an easy to use primary function, called CUR, was implemented. The input of the function CUR is a two dimensional matrix with column and row names. If any of the column or row names is missing then the index of the dimension is assigned automatically. From the matrix *A* the function CUR calculates the *statistical leverage scores* and the matrices *C*, *U*, and *R*. Importantly, the rank parameter (typically denoted *k*) has a fundamental influence on the resulting leverage scores (and thus on the construction of *C* and *R*), since only the top *k* singular vectors of *A* are used in their calculation. Thus, it should be chosen carefully, based on domain-specific considerations. If no value is supplied for *k*, then *k* is arbitrarily set such that the sum of the top *k* singular values is more then 80% of the sum of all singular values. In our implementation the size of the resulting *C*, *U* and *R* matrices is not determined dynamically based on the error parameter *ε*. Rather the number of columns (*c*) and rows (*r*) to be selected are input parameters and the actual error of the approximation is returned if requested. The outputs of function CUR are stored as slots in a S4 class, called CURobj. In addition, in certain cases we have found that the leverage scores themselves can be used directly, and thus we also provide an implementation that selects the columns or rows without involving any randomness. In particular, this involves using the statistical leverage scores as a "ranking function" rather than as an "importance sampling" distribution, and then deterministically choosing *k* or slightly more than *k* of the the highest leverage columns/rows. Selecting data points or features (for example in gene expression studies) in this way makes the analysis more reproducible and interpretable, although we have found that in some cases the inclusion of random additional columns indeed slightly improves the precision of the approximation.

Several other column selection methods are also implemented in rCUR. These can be selected by the parameter "method".

**random **the original method described in [4] that was outlined above; this is the default.

**exact.num.random **like the default method, but it is guaranteed, that exactly as many rows and columns are selected as requested. (In the random case it is only the expectation value.)

**top.scores **the rows and columns with the highest leverage scores are returned deterministically.

**ortho.top.scores **columns and rows are selected in an iteration based on a factor that combines not just the leverage score but also the orthogonality of the next vector to the already selected subspace.

**highest.ranks **rows and columns with the highest rank of leverage score for some rank parameter are selected. Every possible value is tried up to the value of *k*.

These methods are considered experimental and they provide roughly the same precision as the default method. For certain problems with highly correlated columns/rows one method (ortho.top.score) seems to be very promising. In this way the selection of multiple similar columns/rows, which does not contain new information is avoided, hence the necessary number of columns/rows can be reduced.

To extract the matrices *C*, *U*, *R* and the statistical leverage scores from the object CURobj function, the functions getC, getU, getR and leverage, respectively, may be used. With the function top.leverage, one can get the indexes of the rows or columns with highest leverage scores as the most influential features (genes or samples, respectively). Using these indexes one can get subset of the matrix *A* for further analysis.

To improve efficiency the computation of components that are not used can be switched off. In particular, if the restricted CUR decomposition is required, the parameter *r* can be set to the actual number of rows of *A*. In this case row selection is skipped and *X* can be recovered as *UA*. (The actual matrix multiplication *UA* is not performed by the package.)

In addition, with the function plot.leverage, one can plot the statistical leverage scores themselves, highlighting the largest values and indicating the uniform level directly from CURobj.

For users who would like to test the functionalities of the package on published, real world data sets we incorporated the data used by paper [4] presenting CUR decomposition. This is a subset of a soft tissue tumor data set [13]. The 31 samples of dataset belong to three phenotypes gastrointestinal stromal tumor (GIST), leiomyosarcoma (LEIO) and synovial sarcoma (SARC). For each sample, 5520 gene expression values are stored in matrix *STTm* and annotation information in data frame *STTa*. The original, full dataset [13] is downloadable from the Gene Expression Omnibus database (GSE3443) and from Stanford Microarray Database ( http://smd.stanford.edu).

## Results and discussion

We illustrate the benefits of CUR matrix decompositions and dimension reduction with the rCUR package by comparing it with two different previously-published case studies. In the first, we show that feature selection based on leverage scores can differentiate classes with a performance similar to that of the entire gene set of a microarray. In the second, we show that CUR performs well not only in the separation of classes, but in addition we can get comparable results in trend analysis with a fraction of full feature set. We provide all the code that is neccessary to reproduce these results as *Additional file*1*and Additional file*2

### Case study 1: soft tissue tumor discrimination

**Figure 1:**
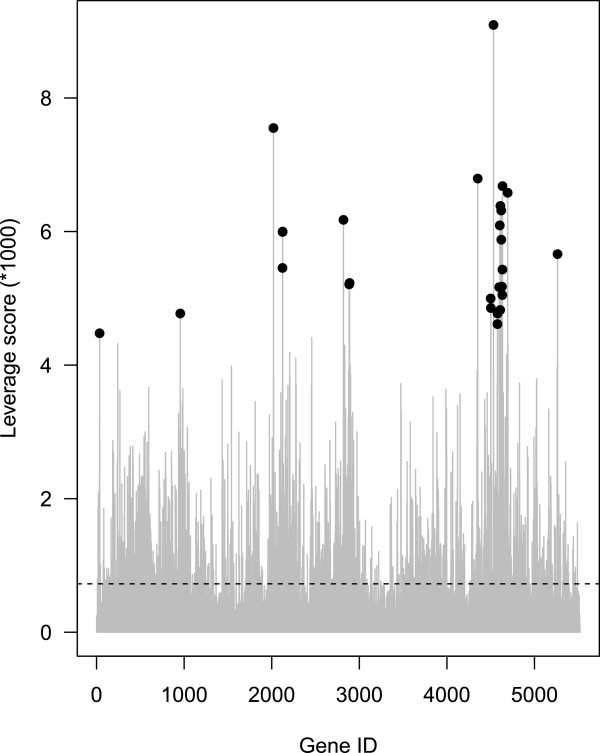
**Feature selection using leverage score.** Normalized leverage scores (grey bars) are presented for each gene (5520) in dataset ordered by row number. The highest 27 leverage scores are dotted with black.

**Figure 2:**
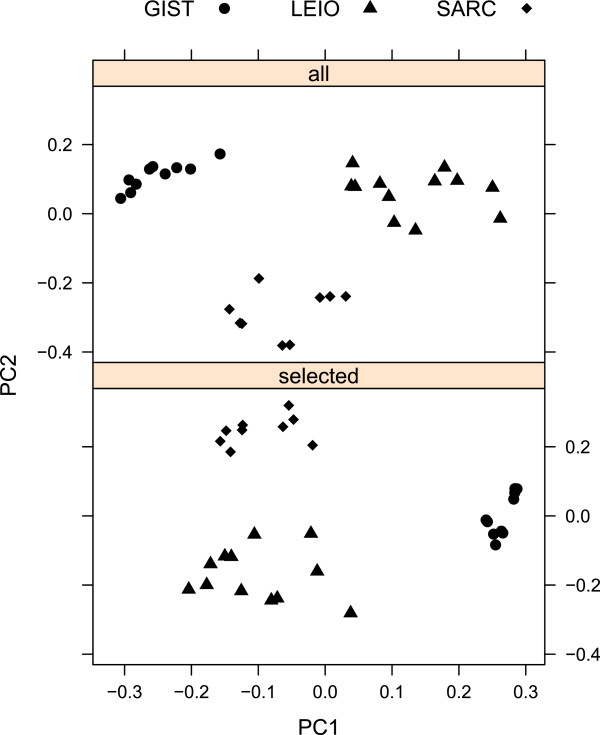
**PCA plots are presented using the first two principal components.** The plot at the top shows PC1 and PC2 using all genes, the one below plots the results based on the selected (27) features. Genes filtered by leverage scores give similar discriminative performance like the whole dataset.

Here our goal is to check if it is possible to separate groups with genes filtered by CUR and obtain a performance similar to that with the total gene set. In this example we use a soft tissue tumor dataset, which is incorporated in the package as mentioned above (STTm, STTa). By using the rCUR package, we repeated the analysis that was performed in the paper publishing the CUR method [4]. After running the function CUR the genes with highest leverage scores are selected as the most influential features (see Figure 1). Then, using the 27 genes with the highest normalized leverage scores, we performed a PCA, just as with the total 5520 genes of dataset. Biplots were created from the two first components from both PCAs (see Figure 2).

According to biplots, one can conclude visually that using CUR as feature selection method we can discriminate the classes with many fewer variables (0.5*%*of the full dataset), obtaining performance comparable to the full set.

### Case study 2: discrimination and trends

**Figure 3:**
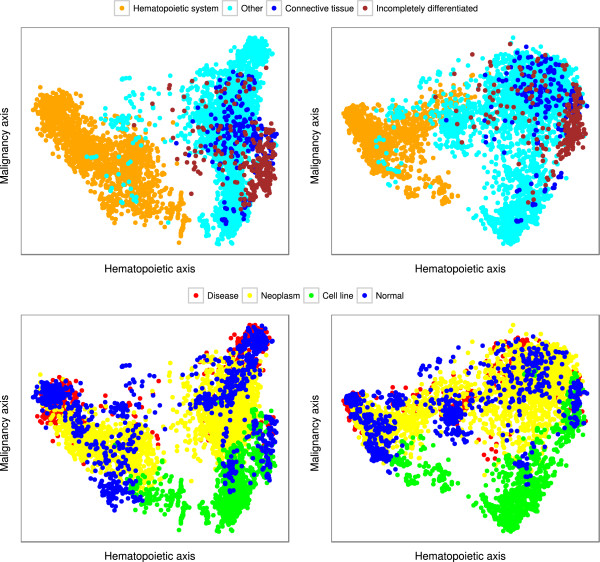
**Trends from principal components.** Based on 5,372 human microarray data Lukk et al. [1] showed that the first two principal components can be interpreted as "hematopoietic" and "malignancy" axes. In their study they used all 22,283 gene expression values in the principal component analysis. The two plots on the left side are reproductions of their results. Classifying the tissue of samples due to a "hematopoietic" direction (hematopoietic system, connective tissue, incompletely differentiated and other) a trend can be found along the horizontal axis (on top). Using another classification of samples (cell line, neoplasm, disease and normal) a "malignancy" trend can be recognized vertically (on bottom). Dots represent the samples colored according to classes determined by Lukk et al. [1]. Using the 250 most influential features filtered by leverage scores from CUR decomposition (with *k *= 5) very similar pattern was plotted (on right).

One of the major problems of microarray studies is that the individual probe values are not always well correlated with the expression of the corresponding gene. On the other hand, it has been shown [1] that even for a very large and inhomogeneous set of microarray samples it is possible to extract reliable information and classify the samples. In that study, the authors collected 5,372 human samples representing 369 different cell and tissue types, disease states, and cell lines. The samples were part of a public international archive, which means that not only the sample parameters but the research groups doing the experiments, the goal of their studies, the sample handling, etc. were very heterogeneous. We have reconstructed their main finding in Figure 3. Note that, despite the mixed origin of the samples, one can clearly identify distinct classes like hematopoietic or malignant samples. After reducing the number of probes from 22,300 to 250 with CUR, we obtain the very same trends in the first few components. Since we have several classes and many dimensions, Figure 3 provides just a visual demonstration of the classification.

**Figure 4:**
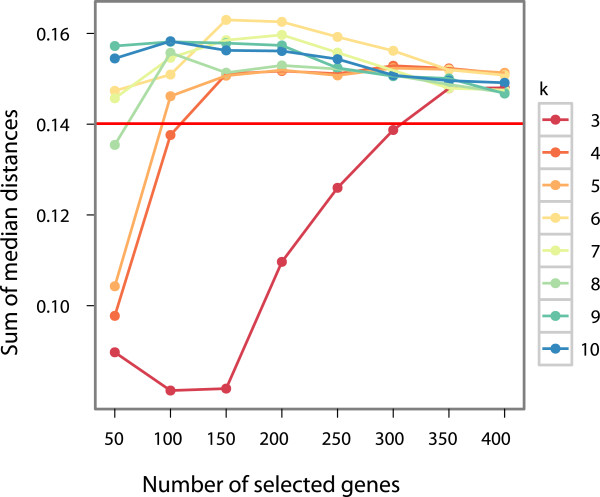
**Measure of separation against the reduced number of genes at different *k *values.** The sum of median distance measure as a function of genes and *k* parameter of CUR (different colors). The horizontal red line shows the baseline value when all the genes are used for PCA. Above 300 genes and *k *= 3 the CUR selected subsets give better separation than the original all-genes and the maximum separation performance is around *k *= 5 using 150 genes.

To make the goodness of the classification more quantitative, we apply the following metrics to measure the separation of the classes. For all group pairs all point pair Euclidean distance was calculated. We measure the separation of two groups by the median of these distances for that group pair. For all group pairs these medians were summarized as a total separation measure. In Figure 4, we plot the value of the measure of separation against the reduced number of genes at different *k* values. We can see that for most of the parameter values the CUR compression not just reproduces the results of the original PCA (horizontal red line) but gives somewhat better values. It is interesting that the critical value where the separation performance of the compressed representation jumps to similar values as the full PCA is *k *= 4 which is the number of the different classes. The optimal separation performance is around 150 genes and *k *= 6, using less than 1% of the original 22,283 genes.

## Conclusions

The package rCUR provides functions for the users to perform CUR matrix decompositions in the R environment. In gene expression studies, it may give an additional way of analysis of differential expression and discriminant gene selection based on the use of statistical leverage scores. The approach proposed [4] is quite novel in the sense of interpretable dimension-reduced matrices and in handling "outliers" as the most important data points. We have also demonstrated that by using rCUR it is possible to significantly reduce the number of necessary probes in classification studies. This may open the way towards much cheaper diagnostic chips.

## Availability and requirements

**Project name:** rCUR

**Project home page:**http://cran.r-project.org/web/packages/rCUR/index.html

**Operating system(s):** Platform independent

**Programming language:** R Other requirements: package MASS, methods, Matrix

**License:** GNU GPL Any restrictions to use by non-academics: none

## End notes

^a^If $C=U_{C}\Sigma_{C}V_{C}^{T}$ is the SVD of *C*, then $C^{+}=V_{C}\Sigma_{C}^{-1}U_{C}^{T}$.

## Competing interests

The authors declare that they have no competing interests.

## Authors’ contributions

AB and NS implemented the functions of rCUR. NS constructed the package, examples and performed all the analysis. ICS, MM and NS wrote the manuscript. All authors read and approved the final manuscript.

## Supplementary Material

Additional file 1 **rCUR package:** The R package rCUR (version 1.1) with functions for CUR decomposition. Click here for file

Additional file 2 **rCUR_Case_Studies.R:** R-script file containing all the sources necessary to reproduce the results presented in the paper.cdqwqC. Click here for file
